# Risk factor evaluation and performance improvement for surgical site infections in patients undergoing abdominal hysterectomy at a large academic safety net hospital

**DOI:** 10.1017/ice.2025.34

**Published:** 2025-05

**Authors:** Anna Buford, Tyler Anderson, Roman Jandarov, Joseph Schaffer, Jacqueline Wells, Marianne Bartlett, Latitia Houston, Calvin White, Laura Buford, Madhuri Sopirala

**Affiliations:** 1 University of Texas Southwestern School of Medicine, Dallas, TX, USA; 2 University of Cincinnati College of Medicine, Cincinnati, OH USA; 3 Obstetrics and Gynecology, University of Texas Southwestern Medical Center, Dallas, TX, USA; 4 Parkland Health, Dallas, TX USA; 5 Division of Infectious Diseases and Geographic Medicine, Department of Internal Medicine, University of Texas Southwestern Medical Center, Dallas, TX, USA

## Abstract

**Objective:**

To identify Surgical Site Infection (SSI) risk factors for abdominal hysterectomy patients and report the results of a performance improvement initiative.

**Design:**

Retrospective case-control.

**Setting:**

Parkland Hospital, an 882-bed academic, safety-net, tertiary referral center and a level 1 trauma center serving a diverse population of primarily uninsured patients in North Texas.

**Participants:**

Patients over 18 who underwent abdominal hysterectomy and were diagnosed with SSIs within 30 days of surgery between 2019 and 2021.

**Methods:**

Cases were matched to controls from the same or closest calendar month in a 1:2 ratio. Chart review of electronic medical records (EMR) was performed comparing variables using Pearson’s χ^2^ test for categorical variables and Student’s t-test for continuous variables followed by logistic regression for multivariate analysis. Upon identifying vaginal preparation technique as an area of improvement while investigating SSI bundle compliance, we implemented an OR staff training intervention.

**Results:**

Diabetes was identified as a significant risk factor while Hispanic or Latino ethnicity was associated with significantly lower rates of infection. Most organisms identified were enteric pathogens. Following the intervention, Parkland’s deep and organ-space Standardized Infection Ratio (SIR) decreased from 1.46 in 2021 to 0.519 for the rolling 12 months as of June 2024.

**Conclusions:**

Our multidisciplinary intervention improving the quality and consistency of pre-operative vaginal preparation was associated with a reduction in abdominal hysterectomy SSI.

## Introduction

Surgical site infections (SSIs) remain a common post-operative complication, affecting an estimated 5% of surgical patients and causing most unplanned hospital readmissions after surgery.^1^ For patients undergoing abdominal hysterectomy, the reported incidence of deep and organ space SSI ranges from 1.1% – 5.7%. Infection rates are higher for abdominal hysterectomy patients enrolled in Medicaid, in addition to higher rates of thromboembolism, myocardial infarction, stroke, pneumonia, and sepsis.^2^ Since 2014, hospitals have publicly reported their rates of SSI after abdominal hysterectomies as part of the Centers for Medicare and Medicaid Services’ (CMS) Hospital-Acquired Condition (HAC) Reduction Program. This program assesses hospital performance across multiple hospital-acquired conditions, and hospitals with higher-than-expected rates of these conditions, including SSI after abdominal hysterectomies, may be subject to reduced reimbursement from CMS.^3^ As a result, the development of evidenced-based surgical care bundles geared toward reducing post-hysterectomy SSIs remains a priority for hospitals reimbursed by CMS. Several studies have shown that bundled prevention methods have significantly lowered surgical site infection rates including those related to hysterectomy.^4–10^

Parkland Hospital is one of the largest safety net hospitals in the United States, serving mostly uninsured and Medicaid-enrolled patients and performing over 1,000 hysterectomies annually. SSI prevention bundles have been in place at Parkland for many years. Our infection prevention (IP) department identified an increasing trend in the incidence of abdominal hysterectomy between 2019 and 2021 compared to the 2015–2018 period (data not shown). In 2021, Parkland’s deep and organ space Standardized Infection Ratio (SIR) of 1.456 in abdominal hysterectomy patients, was consistently above institutional goal of 0.99 for four consecutive quarters in 2021. This study identifies SSI risk factors for abdominal hysterectomy patients at Parkland Hospital and reports our efforts in decreasing SSI in our unique patient population.

## Methods

This is an observational cohort quality improvement study that used historical controls. We also performed a retrospective case-control study that evaluated abdominal hysterectomy recipients. The study was conducted at Parkland Hospital, an 882-bed academic, safety-net, tertiary referral center and a level 1 trauma center serving a diverse population of primarily uninsured patients in North Texas. Evaluation of our SSI revealed that enteric and vaginal pathogens caused hysterectomy SSI in majority of cases where cultures were performed. This led IP to evaluate vaginal preparation technique as an area for improvement. Initial observations of vaginal preparation conducted by IP in March 2022 revealed variations in vaginal preparation technique among OR nursing staff. Despite the absence of baseline data, IP observations revealed inconsistent adherence to the Association of periOperative Registered Nurses (AORN)-recommended vaginal preparation procedure in every case observed during this exploratory phase.^11^ Our findings included moving from dirty to clean area while applying the antiseptic during vaginal preparation and not cleansing the vaginal vault and cervix with antiseptic using a circular motion. In March 2022, we developed a PowerPoint presentation incorporating hospital-specific data and evidence-based rationale to promote adherence to optimal vaginal preparation techniques, and included AORN’s step-by-step guidelines for proper vaginal preparation, to standardize care. Education and in-service were provided to all OR nurses who were involved in care of patients undergoing hysterectomy in March and April 2022. We created a video showing the step-by-step process on a manikin in November 2022. A mandatory return demonstration using a manikin was also performed for the nurses. Weekly audits of pre-operative vaginal preparation technique started in November 2022. On-the-spot education was provided when non-compliance with vaginal preparation protocol was observed. In addition, in-services were conducted at least quarterly and return demonstration using manikin was performed at least annually. In January 2023, staff were educated on the importance of hysterectomy SSI prevention bundle elements already described in literature and were in use at our institution. Posters outlining bundle elements were created and displayed in the OR to reinforce awareness among surgeons, anesthesiologists and nursing staff in May 2023.^12–14^ These bundle elements included maintaining normothermia, blood glucose monitoring for diabetic patients and maintaining blood glucose <200 mg/dL, testing and treating for bacterial vaginosis, preoperative and intraoperative antibiotic prophylaxis, vaginal preparation, abdominal skin preparation, hair clipping guidance and changing gown and gloves when transitioning from vaginal field to abdominal field, changing gloves at the time of facial closure for open hysterectomy cases and utilizing dedicated closure trays for all hysterectomy cases.

We performed a case control study to identify any modifiable risk factors for hysterectomy SSI in our patient population. Cases were defined as patients over 18 who underwent abdominal hysterectomy and were diagnosed with SSI within 30 days of surgery between 2019 and 2021. Cases were matched to controls from the same or closest calendar month in a 1:2 ratio. Data were extracted and manually verified from Parkland electronic medical records (EMR) including surgical reports, laboratory results, pathology reports, and anesthesia logs. Hypothermic cases were defined as cases 15 minutes or shorter in which there was a documented drop in core body temperature below 36°C indicated on intra-operative anesthesia records.

Initial chart review included 18 variables identified as potential risk factors based on the literature. Statistical analysis was performed using Pearson’s χ^2^ test for categorical variables and Student’s t-test for continuous variables followed by logistic regression for multivariate analysis. Variables studied are listed in Table 1. Those that were found on univariate analysis to have contributed to model instability or have *P* > 0.1 or inadequate data were excluded from multivariate analysis.

**Table 1.** Results of univariate analysis for surgical site infection (SSI) risk factors in abdominal hysterectomy cases from 2019-2021 compared to controls

Risk factor	Total number (N = 111)	Total controls (N = 74)	Total SSI cases (N = 37)	p-value*
Age category				
20–29	4	2 (50%)	2 (50%)	0.8571
30–39	26	17 (65.4%)	9 (34.6%)	1.0000
40–49	57	43 (75.4%)	14 (24.6%)	0.0699
50–59	18	9 (50%)	9 (50%)	0.1721
60–69	5	3 (60%)	2 (40%)	1.0000
70–79	1	0 (0%)	1 (100.0%)	0.7225
Race				
White	80	53 (66.3%)	27 (33.8%)	1.0000
** Hispanic**	**66**	50 (75.8%)	16 (24.2%)	**.0241**
Non-Hispanic	14	3 (4.05%)	11 (29.7%)	<.0001
African American	30	21 (70%)	9 (30%)	0.8207
Other race	1	0 (0%)	1 (100%)	0.7225
Current smoker	17	13 (76.5%)	4 (23.5%)	0.5142
**Diabetes mellitus**	**22**	**10 (45.5%)**	**12 (54.5%)**	**0.0353**
ASA Class 3*	**35**	**14 (40.0%)**	**21 (60.0%)**	**0.0001**
Body mass index				
25 and under	11	9 (81.8%)	2 (18.2%)	0.4318
25–30	26	19 (73.1%)	7 (26.9%)	0.5791
30+	74	46 (62.2%)	28 (37.8%)	0.2618
Vaginal preparation				
Betadine	105	72 (68.6%)	33 (31.4%)	0.1817
Chlorhexidine	2	2 (100%)	0 (0%)	0.8008
**None**	**4**	**0 (0%)**	**4 (100%)**	**0.0192**
Preoperative Antibiotics				
**Cefazolin**	**100**	**69 (69%)**	**31 (31%)**	**0.0266**
Cefoxitin	1	1 (100%)	0 (0%)	n/a
Ciprofloxacin	1	0 (0%)	1 (100%)	n/a
Clindamycin + Gentamicin	9	4 (44.4%)	5 (55.6%)	n/a
0 Abx	28	16 (57.1%)	12 (42.9%)	0.3151
1 Abx	76	54 (71.1%)	22 (28.9%)	0.2195
2 Abx	8	4 (50%)	4 (50%)	0.5165
Preoperative steroid	100	70 (70%)	30 (30%)	0.0562
**Open surgery**	**26**	**3 (11.5%)**	**23 (88.5%)**	**0.0000**
Dirty/Infected Wound	2	0 (0%)	2 (100%)	0.2072
Surgery duration > 180 min	71	43 (60.6%)	28 (39.4%)	0.1079
**Hospital stay > 1 day**	**29**	**0 (0%)**	**29 (100%)**	**0.0000**
**Blood loss > 500 mL**	**13**	**0 (0%)**	**13 (100%)**	**0.0000**
Transfusions				
** Perioperative**	**9**	**0 (0%)**	**9 (100%)**	**0.0000**
Postoperative	2	0 (0%)	2 (100%)	0.2072
Postoperative > 4 units of packed red blood cells	1	0 (0%)	1 (100%)	n/a
Temperature				
Ever above 36°C	90	60 (66.7%)	30 (33.3%)	1.0000
Ever below 36°C	99	67 (67.7%)	32 (32.3%)	0.7458
Postoperative glucose				
Under 200	25	4 (16%)	21 (84%)	0.3583
200–250	8	3 (37.5%)	5 (62.5%)	0.4862
250+	2	0 (0%)	2 (100%)	1.0000
Uterine weight > 250g	49	30 (61.2%)	19 (38.8%)	0.3796

*American Society of Anesthesiologists Physical Status Classification System.

All abdominal hysterectomy SSI surveillance was conducted using National Healthcare Safety Network (NHSN).^15^ The project was deemed as quality improvement by an institutional review process and the need for research approval was waived.

## Results

Chart review of all 2019–2021 cases revealed that among the cases where cultures were performed, pathogens were both enteric and vaginal organisms, with *Escherichia coli* (22%/n = 3), *Gardnerella vaginalis* (15%/n = 2), *Staphylococcus aureus* (14%/n = 2) and gram-positive coccus (14%/n = 2) being the most common. Gram-negative bacillus (7%/n = 1), gram-positive bacillus (7%/n = 1), *Enterobacter cloacae* complex (7%/n = 1), Diphtheroids (7%/n = 1), and *Finegoldia magna* (7%/n = 1) were also identified. Many cultures were polymicrobial.

**Figure 1.**
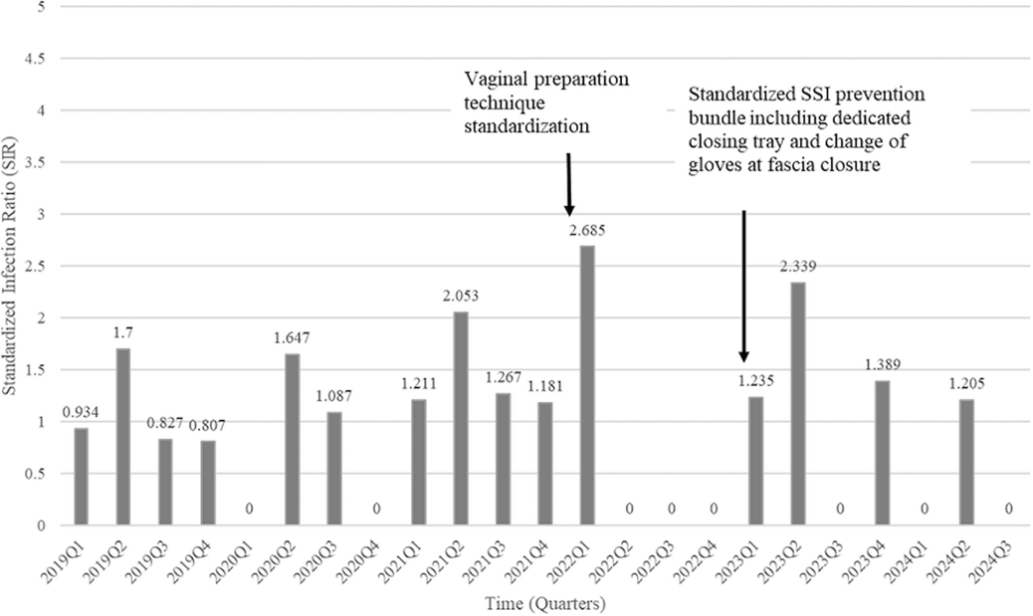
Abdominal hysterectomy deep and organ space SIR.

**Figure 2.**
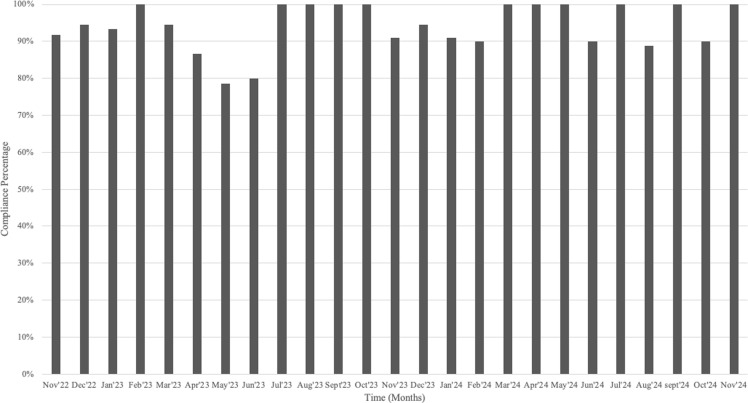
Compliance with standardized vaginal preparation technique.

We designed and implemented an intervention focused on improving preoperative vaginal preparation technique based on our observations that despite being performed for all elective gynecologic surgeries, technique with vaginal preparation was inconsistent (described in methods). After the intervention started, Parkland’s SIR for all hysterectomies decreased from 1.21 in 2021 to 0.76 in 2022. Moreover, Parkland’s Deep and Organ Space SIR decreased from 1.46 to 0.63 over the same period (Figure 1). Though we saw an increased SIR for all post-hysterectomy SSIs along with post-hysterectomy deep and organ space infections in 2023 (1.33 and 1.30, respectively), we continued our efforts in education, case reviews, and monitoring. Compliance with standardized vaginal prep technique is shown in Figure 2. We struggled with compliance with the use of closure tray as per anecdotal reports from nursing (data not available). All post-hysterectomy SSI SIR decreased to 0.765 and deep and organ-space SIR decreased to 0.519 for the rolling 12 months as of June 2024. Deep and organ-space SIR was 0.48 from January to September 2024 (Figure 1).

37 SSI cases from 2019–2021 were compared to 74 controls. Nine variables were identified to be significant on univariate analysis (Table 1). Diabetes (*P* = 0.0353), American Society of Anesthesiologists Physical Status Classification System (ASA) class 3 (p= 0.0001), no vaginal preparation (*P* = 0.0192), open surgery (*P* = 0.0000), hospital stay longer than one day (*P* = 0.0000), blood loss greater than 500 mL (*P* = 0.0000), and perioperative blood transfusion (*P* = 0.0000) were associated with increased infection risk. Hispanic or Latinx ethnicity (*P* = 0.0241) and preoperative Cefazolin prophylaxis (0.0266) were found to be protective. All variables with p-values <=0.1 from the univariate analysis were initially included in the model for multivariate analysis. Some variables demonstrated unstable coefficient estimates and p-values and therefore were removed from the model. Table 2 demonstrates results of multivariate analysis after removing the variables that were causing this instability. Multivariate analysis revealed diabetes (OR, 3.89; 95% CI,1.25–12.08; *P* = 0.0188) as a significant risk factor. Hispanic or Latinx ethnicity was associated with significantly lower rates of infection (OR, 0.31; 95% CI,0.12–0.80; *P* = 0.0160). Of note, the data did not provide sufficient information to quantify the relationship between vaginal preparation and the SSI, while controlling for other variables in the model.

**Table 2.** Independent risk factors for surgical site infection (SSI) cases 2019–2021 included in multivariate analysis

Risk factor	Adjusted OR (95% confidence interval)	p-value
Age category: 40–49	0.66 (0.26–1.65)	0.3696
Ethnicity: Hispanic or Latino	0.31 (0.12–0.80)	0.0160
Diabetes mellitus	3.89 (1.25–12.08)	0.0188
Vaginal preparation: None	Coefficient not estimable	N/A
Preoperative steroid	0.46 (0.10–2.12)	0.3215

## Discussion

While we found vaginal preparation to be a significant risk factor on univariate analysis, this variable was non-estimable in our multivariate analysis. However, the discovery of enteric and vaginal pathogens causing most of the SSI during chart review of cases resulted in audits of pre-operative vaginal preparations, which showed opportunities for improvement with vaginal preparation technique. These findings resulted in hands-on training with OR nursing staff using a manikin in addition to ongoing audits of vaginal preparation technique. This intervention was associated with a decrease in incidence of deep and organ space SSI. Though we saw an increased SIR in 2023, we continued our efforts in education, case reviews, and monitoring. In addition to standardizing our vaginal preparation technique, we also reinforced education on already existent bundle elements including a dedicated closure tray, the practice of changing gloves at the time of fascial closure and changing gown and gloves while moving from vaginal field to abdominal field at the beginning of 2023. We struggled with compliance with the use of closure tray and are in the process of building an electronic monitoring report to obtain ongoing data from the electronic medical record. Our SSI decreased following the vaginal preparation technique intervention. Our audit data shows that the compliance with proper vaginal preparation technique decreased in second quarter of 2023 during which time we had two SSI. Compliance increased during the subsequent months. Our SSI reduced to an SIR of 0.48 from January to September 2024. Although there were some variables that were significant in univariate analysis such as open surgery, hospital stay >1 day, blood loss, and perioperative blood transfusion, our chart reviews did not indicate these were modifiable. We decided to focus our intervention on standardizing vaginal preparation technique and reinforcing the SSI prevention bundle.

Contrasting previous literature, our analysis showed Hispanic ethnicity to be a protective factor from SSI in both univariate and multivariate analysis. These findings may be explained by Parkland Hospital’s patient population, as 59.5% of our study population was Hispanic. In contrast, studies citing no significant racial differences in SSI risk among abdominal hysterectomy patients^16^ reported study populations that are as low as 14% Hispanic. Moreover, our findings that Hispanic ethnicity was associated with reduced SSI risk in our patient population aligns with the frequently reported findings that Black women (who comprised only 27% of our population but 30% of cases) undergo a higher proportion of open hysterectomy and experience more major and minor post-hysterectomy complications compared to White women even when adjusting for confounders.^17^ Our quality improvement initiative reduced hysterectomy surgical site infections (SSIs) by standardizing vaginal preparation technique and reinforcing preventive bundle elements. However, we recognize that social determinants of health (SDOH) may have influenced our results, given our diverse patient population and the unique socioeconomic challenges they face. To address these SDOH factors, we are developing targeted patient education materials to promote adherence to pre- and postoperative instructions. Additionally, we are creating a compliance monitoring report to track patient compliance with preventive measures, enabling us to identify and address areas for improvement. By acknowledging the interplay between SDOH factors and our quality improvement initiative, we can better address the complex needs of our patient population and drive continued improvements in patient outcomes.

Diabetes was a significant risk factor on both univariate and multivariate analysis. This finding is consistent with well-established knowledge that diabetic patients are predisposed to bacterial infection, secondarily to impaired leukocyte activity and reduced chemotaxis and oxidative potential in neutrophils.^18–21^ Our ability to assess the association between postoperative glucose and infection in our study was limited by the fact that post-operative blood glucose was only recorded in 35 patients from our abdominal hysterectomy cohort.

While our vaginal preparation intervention was associated with a reduced SSI incidence, we recognized that sustained infection reduction requires optimization of our entire hysterectomy SSI bundle. We formed a multidisciplinary team with gynecologic surgeons, OR nursing, anesthesiology, infection prevention, performance improvement experts and information technology in October 2022 to optimize and standardize our SSI bundle for long-lasting improvement. Though SSI prevention bundle has been in place at our institution for many years, there were variations in practice. We noted that our increased SIR in 2023 was a result of infections occurring in high-risk surgeries performed by non-General Gynecology groups such as Gynecologic Oncology and Urology in addition to the two SSI in cases performed by the Gynecology group that coincided with a decrease in compliance with proper vaginal preparation technique. We are currently working on expanding our efforts to other specialties that perform high-risk hysterectomies.

Our investigation was conducted at one of the largest safety-net hospitals in the nation, serving a diverse patient population with varying access to healthcare. Identifying modifiable risk factors for SSI in an exclusively safety-net population enables our results to be extrapolated to other large safety-net hospitals serving diverse and vulnerable patient populations. However, our study is not without limitations. First, our work was conducted at a single institution and via retrospective chart review, limiting us to drawing associations between variables. Second, although we initially assessed our SSI prevention bundle elements by doing a walkthrough with surgical and nursing staff, we did not monitor ongoing compliance. This hindered our ability to determine the relative contribution of individual bundle elements to the observed reduction in SIR. Notably, the improvement in SIR preceded our efforts to standardize practice regarding the bundle and coincided with implementation of intervention that addressed vaginal preparation technique. Finally, since our study was conducted at a safety-net hospital that serves an underserved population, we may face distinct challenges that differ from those in other hospitals. Although rooted in a specific hospital setting, our findings and strategies have broader applicability to other healthcare institutions, particularly those serving diverse patient populations. We demonstrate that effective SSI prevention strategies can be implemented in resource-constrained settings and share practical lessons learned from our experience. By sharing our findings, we hope to contribute to the broader conversation on SSI prevention, health disparities, and quality improvement.

In conclusion, this study analyzed modifiable risk factors for SSIs in patients undergoing abdominal hysterectomy at Parkland Hospital. Hospitals should also perform observations to examine techniques used by hospital staff in complying with SSI prevention bundle elements such as preoperative preparation of patients even if documentation does not suggest gaps in care. Based on our findings during OR observations, we conducted a multidisciplinary intervention improving the quality and consistency of pre-operative vaginal preparation in conjunction with the hospital’s already-employed SSI bundle. Our efforts were associated with a decreased SIR in deep and organ-space infections.
